# *Enterococcus faecium* WEFA23-Derived Surface Layer Protein OTC Prevents *Listeria monocytogenes* Invasion by Strengthening Intestinal Barrier Function and Modulating Immune Responses

**DOI:** 10.3390/foods14234110

**Published:** 2025-11-30

**Authors:** Yao He, Bing Dong, Ke Xie, Yingsheng Hu, Yina Huang, Xueying Tao, Hua Wei

**Affiliations:** 1 State Key Laboratory of Food Science and Resources, Nanchang University, Nanchang 330047, China; hy18779168236@163.com (Y.H.);; 2 College of Biological and Food Engineering, Anhui Polytechnic University, Wuhu 241000, China; 3 International Institute of Food Innovation Co., Ltd., Nanchang University, Nanchang 330052, China

**Keywords:** *Enterococcus faecium* WEFA23, OTC protein, *Listeria monocytogenes*, anti-invasion, tight junction, inflammation modulation

## Abstract

*Listeria monocytogenes* (*L. monocytogenes*) is a major foodborne pathogen which can invade intestinal epithelial cells and cause severe systemic infection. Probiotics, as well as their surface layer proteins, hold broad promise for enhancing intestinal barrier function and defending against pathogenic invasion. In the present study, the antagonistic effects of surface layer protein ornithine carbamoyltransferase (OTC) from *Enterococcus faecium* (*E. faecium*) WEFA23 against *L. monocytogenes* were systematically evaluated in vitro in human intestinal epithelial Caco-2 cells, including assessments of anti-adhesion and anti-invasion capacity, inflammatory cytokine responses, intestinal barrier integrity, and transcriptomic changes, by comparing the effects of wild-type *E. faecium* WEFA23 and a previously constructed *E. faecium* WEFA23 *otc* gene knockout strain (*E. faecium* WEFA23 *otc*^−/−^). The results demonstrated that *E. faecium* WEFA23 achieved significant stronger anti-adhesion and anti-invasion capacity of *L. monocytogenes* (*p* < 0.05) in the presence of OTC, potentially through increasing tight junction protein expression, regulating inflammatory cytokines, and modulating the virulence factors of the pathogen. To elucidate the potential mechanism of the inhibitory effect of OTC protein, RNA-seq was performed. The results revealed that the significantly regulated core differentially expressed genes (DEGs), including *ADCY2*, *OARI3*, *CCL5*, and *CXCL9*, were found to be involved in γ-aminobutyric acid (GABA)-ergic synapse, calcium, and toll-like receptor signaling pathways. These findings demonstrated that OTC is involved in blocking *Listeria* invasion and revealed the function of the OTC from *E. faecium* WEFA23 in antimicrobial and intestinal mucosal defense, providing a conceptual foundation for the development of new probiotic intervention strategies in anti-infection.

## 1. Introduction

The intestinal epithelium forms an essential defense that limits the passage of pathogens and toxins while facilitating nutrient absorption and immune surveillance [1]. Disruption of this barrier is a hallmark of many gastrointestinal disorders, facilitating the invasion of foodborne pathogens such as *Listeria monocytogenes* (*L. monocytogenes*). *L. monocytogenes* is a Gram-positive intracellular bacterium leading to listeriosis, a severe foodborne illness characterized by high hospitalization and mortality rates, especially among individuals with compromised immunity, pregnant women, and older adults [2]. Upon ingestion, *L. monocytogenes* is capable of attaching to and penetrating intestinal epithelial cells, which subsequently activates inflammatory responses, compromises tight junction integrity, and thus results in systemic dissemination [3]. Therefore, preventing adhesion and invasion of *L. monocytogenes* to intestinal epithelial cells is a key strategy to mitigate infection and maintain gut barrier function.

Lactic acid bacteria have gained growing interest for their capacity to counteract the effects of pathogens through mechanisms including competitive inhibition of pathogen adhesion, modulation of host immune responses, and strengthening tight junctions in intestinal epithelial cells [4]. However, accumulating evidence suggested that these effects were not only attributable to the presence of live bacteria. Instead, probiotic-derived metabolites and surface-associated components, e.g., surface layer proteins (SLPs), exopolysaccharides, and organic acids, were emerging as key effectors in modulating host–pathogen interactions [5]. Among these, several *Enterococcus* strains have been reported to inhibit *L. monocytogenes* through the production of enterocins, enhancement of epithelial barrier function, and competition for adhesion sites on the intestinal mucosa. For example, *Enterococcus faecium* (*E. faecium*) LCW 44 substantially reduced *L. monocytogenes* adhesion to Caco-2 and HT-29 cells [6]. Besides live bacteria, heat-killed *E. faecium* BGPAS1-3 was shown to protect tight junction integrity in Caco-2 monolayers infected with *L. monocytogenes*, preventing barrier disruption [7]. Moreover, bacteriocin-like substances (e.g., enterocin P) produced by certain *Enterococcus* strains show potent antimicrobial activity against *L. monocytogenes* [8]. Therefore, identifying and characterizing specific probiotic-derived factors responsible for anti-pathogen activity and clarifying the underlying mechanism have become critical focuses in probiotic research.

Previous studies identified *E. faecium* WEFA23, a strain isolated from the gastrointestinal tract of a healthy newborn infant, along with its extracted crude SLPs, which exhibited strong antagonistic activity against *L. monocytogenes* in vitro and in vivo [9,10]. Further, ornithine carbamoyltransferase (OTC) was identified as a key component of SLPs via liquid chromatography–Tandem mass spectrometry (LC-MS/MS) and exerted ameliorating effects on *L. monocytogenes*-induced inflammation via Toll-like receptor 2 (TLR2)-mediated nuclear factor kappa B (NF-κB) and mitogen-activated protein kinase (MAPK) signaling in RAW 264.7 cells [11]. Notably, OTC has also been recognized as a component of surface layer proteins in *Clostridium perfringens* ATCC 13124 and *Streptococcus suis*, where it is associated with biofilm formation, adhesion, and immunogenicity [12,13,14]. However, the precise role of the OTC protein in mediating the anti-*Listeria* effects of *E. faecium* WEFA23 against *L. monocytogenes* in the intestinal cells remains unclear.

In this study, the role of *E. faecium* WEFA23 OTC in antagonizing *L. monocytogenes* infection in Caco-2 intestinal epithelial cells was investigated by comparing the effect of *E. faecium* WEFA23 and the *otc* gene deletion mutant (*E. faecium* WEFA23 *otc*^−/−^), in terms of anti-adhesion, anti-invasion ability, cytokines, tight junction proteins and virulence factor expression, and transcriptome changes. To date, this study provides novel evidence that OTC is essential for mediating the protective effects of *E. faecium* WEFA23 against *L. monocytogenes*, and offers new insights into the molecular interplay between probiotics, host cells, and pathogens.

## 2. Materials and Methods

### 2.1. Materials

Brain Heart Infusion (BHI), Luria–Bertani (LB), and PALCAM agar were purchased from Hopebio Biotechnology Co., Ltd. (Qingdao, Shandong, China). Dulbecco’s Modified Eagle’s medium (DMEM) and TRIzol reagent were purchased from Servicebio Co., Ltd. (Wuhan, Hubei, China). Heat-inactivated fetal bovine serum (FBS) was purchased from Cellmax Technology Co., Ltd. (Beijing, China). Triton X-100 and gentamicin were purchased from Solarbio Technology Co., Ltd. (Beijing, China). Reverse transcription system kit and SYBR Premix Ex Taq II were purchased from Takara Bio-Tech (Beijing) Co., Ltd. (Beijing, China). The primers for real-time quantitative PCR were synthesized by Bioengineering (Shanghai) Co., Ltd. (Shanghai, China). Enzyme-linked immunosorbent assay (ELISA) kits for tight junction proteins test were purchased from Yansheng Biology Technology Co., Ltd. (Shanghai, China). All other chemicals were of analytical grade.

### 2.2. Bacterial Strains and Cell Culture Conditions

*E. faecium* WEFA23, originally isolated from a healthy newborn infant and deposited in the China Center for Type Culture Collection with strain number CCTCC NO. M201578520151228, was cultured in BHI agar or broth under aerobic conditions at 37 °C for 12 h. The construction and validation of *E. faecium* WEFA23 *otc*^−/−^ were performed as previously described [11]. Briefly, flanking fragments of the *otc* gene were cloned into a suicide vector, introduced into *E. faecium* WEFA23, and the deletion was confirmed by sequencing and quantitative PCR. The mutant strain was also cultured under the same conditions as the *E. faecium* WEFA23. *L. monocytogenes* CMCC54007 was grown in LB broth at 37 °C for 14 h.

The human intestinal epithelial cell line Caco-2 was obtained from the Cell Bank of the Chinese Academy of Sciences. Cells were maintained in DMEM containing 10% (*v*/*v*) heat-inactivated FBS, 100 U/mL penicillin and 100 μg/mL streptomycin at 37 °C in a humidified incubator with 5% CO_2_ (*v*/*v*). The medium was replaced twice daily until the monolayers reached approximately 80–90% confluence, after which the cells were rinsed twice with sterile PBS (pH 7.4) prior to subsequent experiments.

### 2.3. Anti-Adhesion Assay

The assay evaluating the ability of probiotics to inhibit pathogen adhesion to intestinal cells was conducted according to previously reported methods, with minor modifications [15]. Briefly, Caco-2 cells (approximately 1 × 10^5^ per well) were seeded into 12-well plates and cultured for 18 h. Then 10^7^ CFU probiotics (*E. faecium* WEFA23 or *E. faecium* WEFA23 *otc*^−/−^) were added to incubate with Caco-2 cells for 1 h, following which 10^7^ CFU *L. monocytogenes* CMCC54007 were added for another 1 h. Wells with only pathogens were added to serve as the control. Following incubation, non-adherent bacteria were removed by washing the monolayers four times with sterile PBS. The Caco-2 cells were then detached using an EDTA–trypsin solution, and the resulting cell suspensions were serially diluted and plated onto PALCAM agar for enumeration. The inhibitory effect on *L. monocytogenes* adhesion was evaluated by comparing bacterial attachment in the presence of probiotics (*E. faecium* WEFA23 or its *otc*-deficient mutant) with that observed for *L. monocytogenes* alone. The inhibition rate was calculated as: inhibition of adhesion = (1 − T_1_/T_2_), where T_1_ and T_2_ represent the percentages of adherent *L. monocytogenes* in the presence and absence of the probiotic strains (*E. faecium* WEFA23 or its *otc*-deficient mutant), respectively.

### 2.4. Anti-Invasion Assay

The assay evaluating the ability of probiotics to inhibit pathogen invasion into intestinal epithelial cells was adapted from previously described methods, with minor modifications [16]. Briefly, Caco-2 cells (approximately 1 × 10^5^ per well) were seeded into 12-well plates and incubated for 18 h. Subsequently, 1 × 10^7^ CFU *E. faecium* WEFA23 or *E. faecium* WEFA23 *otc*^−/−^ were added in DMEM and co-cultured with the cells at 37 °C in 5% CO_2_ for 1 h. This was followed by the addition of 1 × 10^7^ CFU of *L. monocytogenes* CMCC54007 under the same conditions for another 1 h. After co-infection, the well was subsequently covered with DMEM containing 100 μg/mL gentamicin and 50 μg/mL streptomycin for 2 h to remove the extracellular bacteria. The monolayers were then lysed with 0.1% Triton X-100 for 3 min, and the released intracellular *L. monocytogenes* were serially diluted and plated onto PALCAM agar for enumeration.

### 2.5. mRNA Level Determination of Cytokines, Tight Junction Proteins, and Virulence Factors

Total RNA was isolated using TRIzol reagent, and its integrity, concentration, and purity were determined with a NanoDrop 2000 spectrophotometer (Thermo Fisher Scientific, Waltham, MA, USA) prior to reverse transcription. First-strand cDNA was synthesized using a PrimeScript™ 1st Strand cDNA Synthesis Kit (6210A, Takara) following the manufacturer’s protocol. mRNA expression levels were quantified by reverse transcription quantitative polymerase chain reaction (RT-qPCR) with SYBR Premix Ex Taq II (RR420A, Takara). Each reaction was performed in triplicate, and data were analyzed using the 2^−ΔΔCt^ method. The primer sequences used for RT-qPCR are listed in Table 1.

### 2.6. Protein Level Measurement of Tight Junction Protein

The protein levels of tight junction proteins [Claudin-1 (YS-S388, Yansheng), Occludin (YS-S11620, Yansheng), and ZO-1 (YS-S351, Yansheng)] were measured in cell culture using ELISA kits according to the manufacturer’s instructions.

### 2.7. RNA Sequencing

Total RNA was isolated from Caco-2 cells using TRIzol^®^ Reagent following standard procedures. RNA integrity and purity were determined with an Agilent 5300 Bioanalyzer (Agilent Technologies, Santa Clara, CA, USA) and quantified using a NanoDrop ND-2000 spectrophotometer. Samples that met the quality requirements (OD_260/280_ = 1.8–2.2, OD_260/230_ ≥ 2.0, RQN ≥ 6.5, 28S/18S ≥ 1.0, and RNA amount > 1 μg) were used for library construction.

RNA purification, cDNA synthesis, library preparation, and sequencing were carried out by Shanghai Majorbio Bio-Pharm Biotechnology Co., Ltd. (Shanghai, China). mRNA-seq libraries were generated using the Illumina^®^ Stranded mRNA Prep, Ligation kit from 1 μg of total RNA. Polyadenylated RNA was enriched using oligo (dT) magnetic beads and subsequently fragmented. First- and second-strand cDNA synthesis was performed with a SuperScript double-stranded cDNA synthesis kit (Invitrogen, Carlsbad, CA, USA) using random hexamer primers. The resulting cDNA was subjected to end-repair, phosphorylation, adapter ligation, and size selection to obtain ~300 bp fragments. Libraries were amplified with Phusion high-fidelity DNA polymerase (NEB) for 15 cycles and quantified using a Qubit 4.0 fluorometer, Thermo Fisher Scientific, Waltham, MA, USA.

Sequencing was performed on either the Illumina NovaSeq X Plus platform (PE150) (Illumina, Inc., San Diego, CA, USA) with the NovaSeq Reagent Kit or the DNBSEQ-T7 platform (PE150) with the DNBSEQ-T7RS Reagent Kit (FCL PE150 v3.0).

### 2.8. Statistical Analysis

Statistical analyses were performed using GraphPad Prism 8 (GraphPad Software, San Diego, CA, USA). Depending on the data distribution and experimental design, the Kruskal–Wallis test, one-way ANOVA, or two-way ANOVA was applied. Post hoc comparisons were conducted with Tukey’s test. Data are presented as mean ± standard deviation (SD), and differences were considered statistically significant at *p* < 0.05.

## 3. Results

### 3.1. Effect of OTC on the Adhesive and Invasive Capacity of L. monocytogenes in Caco-2 Cells

**Figure 1:**
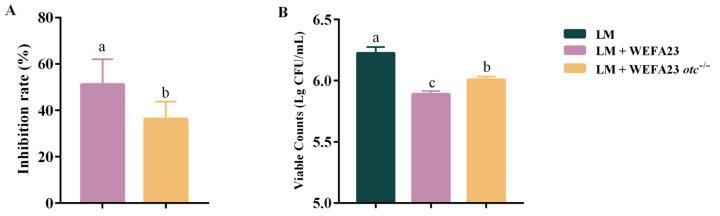
Inhibitory effects of *E. faecium* WEFA23 and its mutant strain *E. faecium* WEFA23 *otc*^−/−^ on adhesion and internalization of *L. monocytogenes* CMCC54007 in Caco-2 cells. (**A**) Anti-inhibition rate, and (**B**) viable bacteria counts of *L. monocytogenes* CMCC54007 after lysis by Triton X-100. LM, *L. monocytogenes*; WEFA23, *E. faecium* WEFA23; WEFA23 *otc*^−/−^, *otc* gene deletion mutant of *E. faecium* WEFA23; CFU, colony-forming units; Lg, log_10_ transformation. Different letters indicate significant differences among groups (*p* < 0.05).

The initial step of pathogenic bacterial infection in the host is adhesion to and invasion into intestinal epithelial cells; therefore, the anti-adhesion and anti-invasion ability of *E. faecium* WEFA23 and its mutant strain with OTC deficiency (*E. faecium* WEFA23 *otc*^−/−^) against *L. monocytogenes* in intestinal epithelial cells Caco-2 was detected. As shown in Figure 1A, deficiency of OTC significantly decreased the anti-adhesion ability of the strain to *L. monocytogenes* (*p* < 0.05). As for anti-invasion capacity revealed in Figure 1B, both *E. faecium* WEFA23 and the mutant with OTC deficiency could significantly decrease the bacteria count invaded into the Caco-2 cells compared to that of the model group (*p* < 0.001), while the mutant intervention showed significantly higher bacteria loads of Lg 5.63 ± 0.10 paralleled by that of Lg 6.02 ± 0.16 (*p* < 0.05) with *E. faecium* WEFA23 intervention. These results revealed the critical role of OTC in the antagonism activity of *E. faecium* WEFA23 to *L. monocytogenes* in intestinal cells.

### 3.2. Effect of OTC on the mRNA Level of Cytokines in Caco-2 Cells

**Figure 2:**
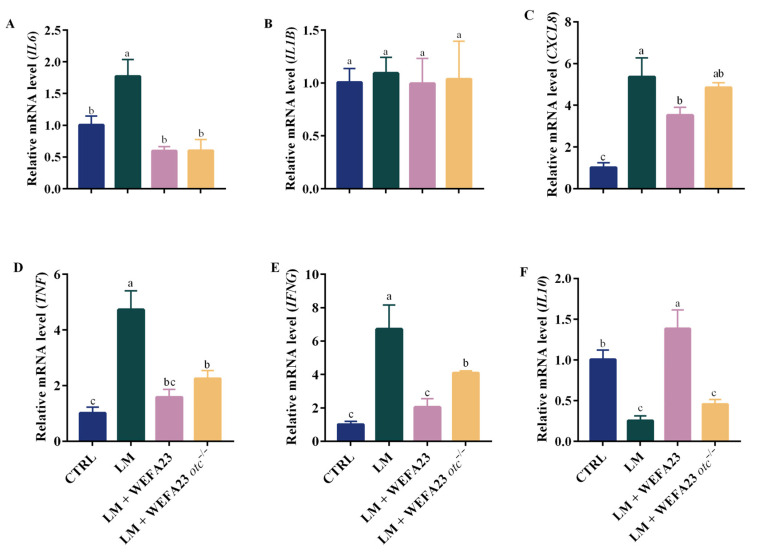
Effects of *E. faecium* WEFA23 and its mutant strain *E. faecium* WEFA23 *otc*^−/−^ on the transcriptional expression of cytokines in *L. monocytogenes* CMCC54007-treated Caco-2 cells. mRNA levels of (**A**) *IL6*, (**B**) *IL1B*, (**C**) *IL8*, (**D**) *TNF*, (**E**) *IFNG*, and (**F**) *IL10.* CTRL, blank control; LM, *L. monocytogenes*; WEFA23, *E. faecium* WEFA23; WEFA23 *otc*^−/−^, *otc* gene deletion mutant of *E. faecium* WEFA23; *IL6*, interleukin-6; *IL1B*, interleukin-1 beta; *CXCL8*, C-X-C motif chemokine ligand 8; *TNF*, tumor necrosis factor-α; *IFNG*, interferon-γ; *IL10*, interleukin-10. Different letters indicate significant differences among groups (*p* < 0.05).

Invading pathogenic bacteria in intestinal epithelial cells typically trigger inflammatory responses in the host cells; hence, the mRNA levels of related cytokines were tested. As observed in Figure 2, infection with *L. monocytogenes* led to a significant increase in the mRNA levels of *IL6*, *CXCL8*, *TNF*, and *IFNG* (*p* < 0.01 for *IL6*, *p* < 0.001 for other cytokines), along with a significant decrease in transcription level of *IL10* (*p* < 0.001). Intervention with both *E. faecium* WEFA23 and mutant strain with OTC deficiency could down-regulate the mRNA expression of *IL6*, *TNF*, and *IFNG* significantly (*p* < 0.05 for *IFNG* in LM + *E. faecium* WEFA23 *otc*^−/−^ group, *p* < 0.001 for others), while only intervention with the former could significantly decrease *CXCL8* (*p* < 0.05) and increase *IL10* (*p* < 0.001) mRNA levels. Moreover, there were significant differences in the mRNA expression of *IFNG* (*p* < 0.05) and *IL10* (*p* < 0.001) between the two treatments. Overall, these results indicated that OTC protein could modulate the transcription disorder induced by *L. monocytogenes* infection to some degree.

### 3.3. Effect of OTC on the Tight Junction Integrity in Caco-2 Cells

**Figure 3:**
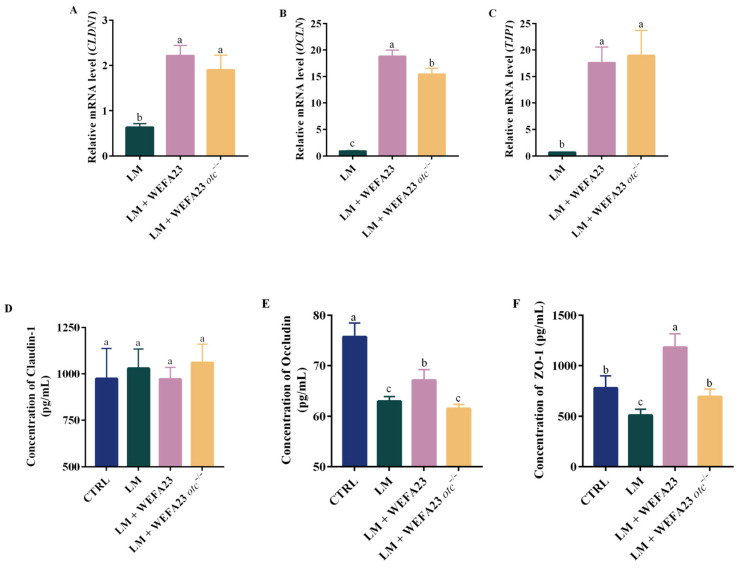
Effects of *E. faecium* WEFA23 and its mutant strain *E. faecium* WEFA23 *otc*^−/−^ on the intestinal barrier function of *L. monocytogenes* CMCC54007-treated Caco-2 cells. mRNA levels of (**A**) *CLDN1*, (**B**) *OCLN*, and (**C**) *TJP1*; concentration of (**D**) Claudin-1, (**E**) Occludin, and (**F**) ZO-1 in Caco-2 cells. CTRL, blank control; LM, *L. monocytogenes*; WEFA23, *E. faecium* WEFA23; WEFA23 *otc*^−/−^, *otc* gene deletion mutant of *E. faecium* WEFA23; *CLDN1*, Claudin-1; *OCLN*, Occludin; *TJP1*, tight junction protein 1. Different letters indicate significant differences among groups (*p* < 0.05).

Pathogenic bacterial infection disrupts intestinal epithelial barrier integrity by impairing tight junctions; herein, both mRNA and protein expression of tight junction proteins of Caco-2 cells were investigated. As shown in Figure 3A–C, infected with *L. monocytogenes* led to down-regulation of *CLDN1*, *OCLN*, and *TJP1* with fold changes of approximately 0.6, 0.9, and 0.7, while treatments with both strains could significantly improve the fold change in TJPs, ranging from 2–18 folds (*p* < 0.01). For the translation level indicated in Figure 3D–F, incubation with the pathogen significantly declined the expression of Occludin (*p* < 0.001) and ZO-1 (*p* < 0.05), which presented significant improvements (*p* < 0.05 for Occludin; *p* < 0.001 for ZO-1) after intervention with *E. faecium* WEFA23. Notably, there were significant differences in the expression of Occludin (*p* < 0.05) and ZO-1 (*p* < 0.01) between the two strain treatments. Collectively, these results demonstrated that OTC protected the tight junction integrity in *L. monocytogenes*-treated Caco-2 cells.

### 3.4. Effect of OTC on the mRNA Levels of Virulence Factors of L. monocytogenes

**Figure 4:**
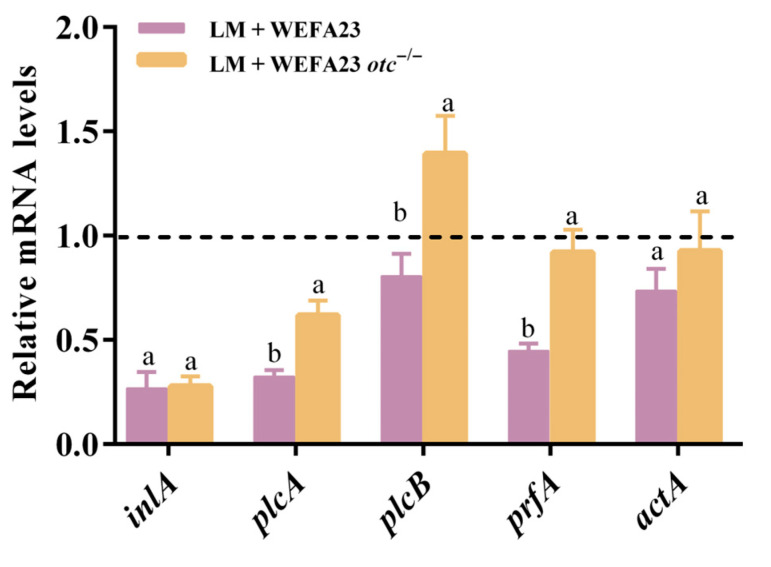
Effects of *E. faecium* WEFA23 and its mutant strain *E. faecium* WEFA23 *otc*^−/−^ on the transcriptional expression of virulence factors of *L. monocytogenes* CMCC54007. WEFA23, *E. faecium* WEFA23; WEFA23 *otc*^−/−^, *otc* gene deletion mutant of *E. faecium* WEFA23; *inlA*, internalin A; *plcA*, phosphatidylinositol-specific phospholipase C; *plcB*, broad-range phospholipase C; *prfA*, positive regulatory factor A; *actA*, actin assembly-inducing protein A. Different letters indicate significant differences among groups (*p* < 0.05).

Invasion of intestinal epithelial cells by *L. monocytogenes* is mediated by its intrinsic virulence factors; thus, the mRNA levels of associated virulence factors after intervention of two strains were evaluated. As observed in Figure 4, treatment with *E. faecium* WEFA23 down-regulated transcription levels of *inlA*, *plcA*, *plcB*, *prfA*, and *actA* to 0.2, 0.3, 0.8, 0.4, and 0.7 folds, while corresponding fold changes in *E. faecium* WEFA23 *otc*^−/−^ achieved 0.2, 0.6, 1.4, 0.9, and 0.9. Additionally, significant differences appeared in *plcA*, *plcB,* and *prfA* (*p* < 0.01 for *plcA*, *p* < 0.001 for the other two) between the two treatments. The results indicated that OTC protein could decrease the mRNA expression of *L. monocytogenes*.

### 3.5. Effect of OTC on the Changes in Transcriptional Profile in Caco-2 Cells

**Figure 5:**
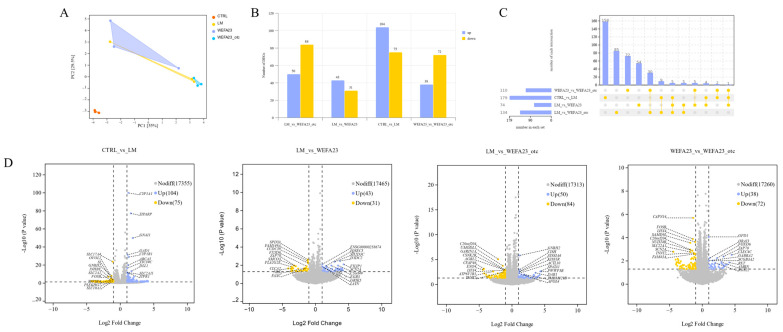
Transcriptomic profiling of *L. monocytogenes* CMCC54007-treated Caco-2 cells following interventions with *E. faecium* WEFA23 and its mutant strain *E. faecium* WEFA23 *otc*^−/−^. (**A**) Principal component analysis (PCA) showing clear separation among the four treatment groups, indicating distinct global transcriptional responses; (**B**) numbers of differentially expressed genes (DEGs) in each pairwise comparison (CTRL vs. LM; LM vs. WEFA23; LM vs. WEFA23_otc; WEFA23 vs. WEFA23_otc), illustrating the extent of transcriptional alterations induced by WEFA23 and the mutant strain; (**C**) UpSet plot depicting the overlap of DEGs among comparison groups, highlighting shared and unique transcriptional signatures associated with WEFA23 and the mutant strain, yellow dots indicate the presence of DEGs in the respective comparison groups, and lines connecting multiple yellow dots represent shared DEGs among those groups; (**D**) volcano plots for the four pairwise comparisons, showing significantly up-regulated and down-regulated genes, with representative DEGs labeled to illustrate major transcriptional changes. CTRL, blank control; LM, *L. monocytogenes*; WEFA23, *E. faecium* WEFA23; WEFA23_otc, *otc* gene deletion mutant of *E. faecium* WEFA23.

To elucidate the underlying mechanisms, transcriptome analysis was conducted using RNA-sequencing to examine the changes in transcriptional profile of *L. monocytogenes*-treated Caco-2 cells under intervention of *E. faecium* WEFA23 and *E. faecium* WEFA23 *otc*^−/−^. Principal component analysis (PCA) (Figure 5A) showed that the CTRL group with biological replicates of strong clustering, while there was some clustering between the other three groups, among which LM and *E. faecium* WEFA23 *otc*^−/−^ demonstrated closer clustering. The bar chart revealed in Figure 5B showed the number of differentially expressed genes (DEGs) across four pairwise comparison groups. A total of 179 DEGs were identified in the CTRL_vs_LM group, including 104 up-regulated and 75 down-regulated genes, indicating a strong host response to *L. monocytogenes* infection. In the LM_vs_WEFA23 comparison, WEFA23 intervention reversed 74 genes (43 up-regulated, 31 down-regulated), whereas the LM_vs_WEFA23_otc group showed 134 DEGs (50 up-regulated, 84 down-regulated), suggesting that OTC deletion altered the regulatory capacity of the strain. Notably, 110 DEGs (38 up-regulated, 72 down-regulated) were observed between WEFA23 and WEFA23_otc treatment groups. An UpSet plot in Figure 5C was generated to visualize the overlap of DEGs across four comparison groups: CTRL_vs_LM, LM_vs_WEFA23, LM_vs_WEFA23_otc, and WEFA23_vs_WEFA23_otc, with 158, 54, 85, and 72 unique genes, respectively, and only 9 genes were common to all groups.

**Figure 6:**
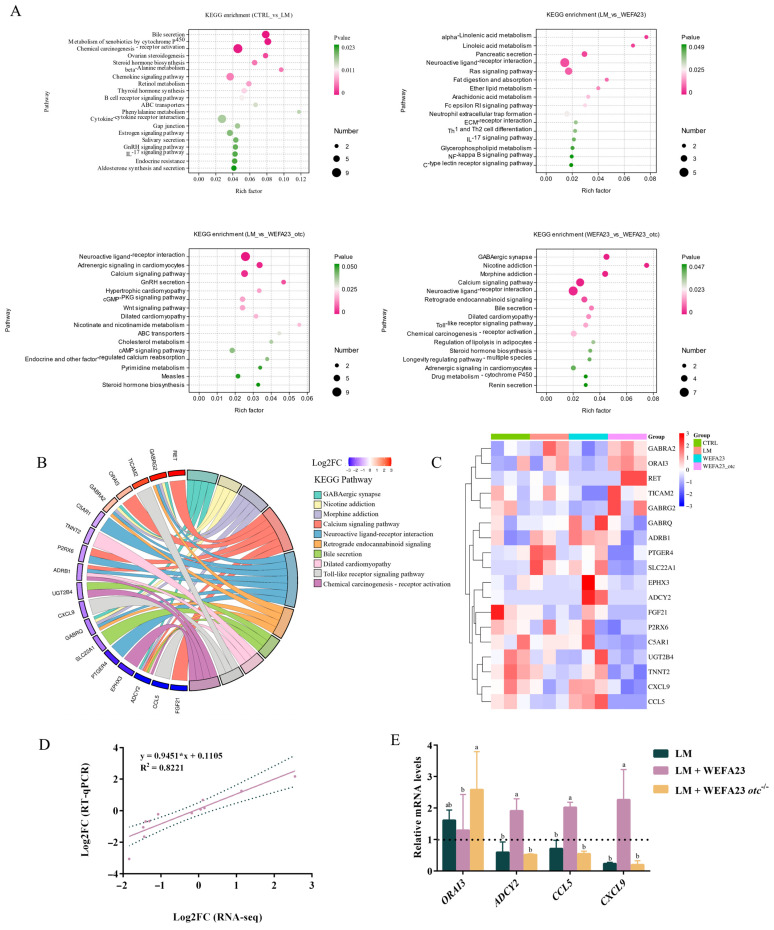
Functional and validation analyses of transcriptomic alterations in *L. monocytogenes* CMCC54007-treated Caco-2 cells following interventions with *E. faecium* WEFA23 and its mutant strain *E. faecium* WEFA23 *otc*^−/−^. (**A**) KEGG pathway enrichment analysis for the four pairwise comparisons (CTRL vs. LM; LM vs. WEFA23; LM vs. WEFA23_otc; WEFA23 vs. WEFA23_otc), highlighting the major biological pathways affected by OTC; (**B**) KEGG chord diagram illustrating the top 10 enriched pathways and their associated genes in the WEFA23 vs. WEFA23_otc comparison, showing the functional connections of key DEGs; (**C**) hierarchical clustering heatmap of significantly dysregulated genes (FDR < 0.05) in the WEFA23 vs. WEFA23_otc group, revealing distinct transcriptional signatures induced by the OTC; (**D**) correlation analysis between RNA-seq and RT-qPCR results, demonstrating consistency in gene expression trends across the two methods; (**E**) RT-qPCR validation of representative genes (*ORAI3*, *ADCY2*, *CCL5*, and *CCL9*) in Caco-2 cells, confirming the transcriptomic findings. CTRL, blank control; LM, *L. monocytogenes*; WEFA23, *E. faecium* WEFA23; WEFA23_otc & WEFA23 *otc*^−/−^, *otc* gene deletion mutant of *E. faecium* WEFA23. Different letters indicate significant differences among groups (*p* < 0.05).

To visualize the overall distribution of DEGs, a volcano plot was generated in Figure 5D, and the results showed that these DEGs were widely distributed. KEGG analysis (Figure 6A) showed that the enriched signaling pathways focused on immune and inflammation-related pathways (IL-17 signaling pathway, cytokine–cytokine receptor interaction, Th1 and Th2 cell differentiation, C-type lectin receptor signaling pathway, and Toll-like receptor signaling pathway), neuroendocrine signaling pathways (neuroactive ligand-receptor interaction, nicotine and nicotinate metabolism, GnRH secretion, GABAergic synapse, adrenergic signaling in cardiomyocytes and morphine addiction), metabolic regulatory pathways (linoleic acid metabolism, alpha-linolenic acid metabolism and glycerophospholipid metabolism) and cell signal transduction pathways (wnt signaling pathway, calcium signaling pathway, ECM-receptor interaction and chemical carcinogenesis—receptor activation). To further dig out the key effector in the anti-*Listeria* activity of OTC of *E. faecium* WEFA23, a chord diagram (Figure 6B) illustrated the interaction between key DEGs and enriched KEGG pathways between *E. faecium* WEFA23 and *E. faecium* WEFA23 *otc*^−/−^ in the top 10 ranked. The results demonstrated that core genes, including *ADCY2*, *GABRA2*, *SLC32A1*, *ADRB1*, *ORAI3*, and *SLC6A3*, were found to be involved in GABAergic synapse, neuroactive ligand-receptor interaction, and calcium signaling pathways. Moreover, *CXCL9* and *CCL5* genes associated with the Toll-like receptor signaling pathway also showed significant regulation between the two interventions. Additionally, Figure 6C showed a clustered heat map that was constructed based on the results of the KEGG enrichment analysis, which visualized the genes involved in the pathways of the top 10 ranked. All in all, these results revealed that OTC protein could change the transcriptional profile in *L. monocytogenes*-treated Caco-2 cells.

To verify the reliability of the RNA-seq data, qRT-PCR was performed on 12 representative DEGs. As observed in Figure 6D, a positive correlation (R^2^ = 0.8221, *p* < 0.001) was demonstrated between RNA-Seq and qRT-PCR results, indicating high consistency between the two datasets and confirming the robustness and accuracy of the transcriptomic analysis. Further, the mRNA expression of some DEGs of interest was investigated, as shown in Figure 6E. Treatment with *L. monocytogenes* led to regulatory disorders in Caco-2 cells. Intervention with *E. faecium* WEFA23 significantly increases mRNA expression of *ADCY2* (*p* < 0.05), *CCL5* (*p* < 0.05), as well as *CXCL9* (*p* < 0.001), and a decreasing trend of *ORAI3*, while deficiency of the OTC abolished the regulatory effect and achieved a result similar to *L. monocytogenes* exposure. These results confirmed that OTC could regulate specific genes in *L. monocytogenes*-treated Caco-2 cells.

## 4. Discussion

*L. monocytogenes* is a highly invasive foodborne pathogen capable of reaching the intestinal barrier, resulting in severe systemic infections, e.g., meningitis, septicemia, and maternal–fetal transmission, particularly in immunocompromised individuals [17]. Given the adhesive and invasive abilities to intestinal epithelial cells, developing effective strategies to prevent *L. monocytogenes* infection at the mucosal level remains a major public health priority. In the current study, the critical role of the surface layer protein OTC from *E. faecium* WEFA23 in antagonizing *L. monocytogenes* invasion in intestinal epithelial cells Caco-2, and the potential mechanism, was systemically investigated.

Adhesion to and subsequent invasion into host cells constitute the initial step in pathogenic bacterial infection [18]; thus, the ability of both wild-type *E. faecium* WEFA23 and its *otc* gene knockout strain on anti-adhesion and anti-invasion of *L. monocytogenes* in Caco-2 cells was investigated. The results showed that deficiency of OTC protein significantly decreased the capacity of *E. faecium* WEFA23 to inhibit the adhesion and invasion of pathogens, indicating the critical role of it in the inhibitory activity of *E. faecium* WEFA23. Choudhary et al. reported that the surface layer protein SlpH from *Lactobacillus helveticus* NCDC 288 markedly reduced the adhesion of enterotoxigenic *Escherichia coli* by 70% and 76%, and that of *Salmonella* Typhimurium SL1344 by 71% and 75%, in exclusion and competition assays using Caco-2 cells [19]. Similar to the work combined with molecular biology methods, Roshila et al. reported that heterologous expression of the *Listeria* adhesion protein (LAP) on the surface of *Lactobacillus casei* significantly enhanced its ability to inhibit the adhesion, invasion, and internalization of *L. monocytogenes* in intestinal cells [20].

Inflammation plays a dual role in pathogen defense and tissue damage. The cytokine patterns in Figure 2 emphasized the central role of OTC in the immunomodulatory activity of *E. faecium* WEFA23 under *L. monocytogenes* infection. The wild-type strain, which expresses OTC, more effectively reduced *CXCL8* and *IFNG* and restored *IL10*, whereas the OTC-deficient mutant showed markedly weaker effects on these cytokines, indicating that OTC contributes specifically to the regulation of major inflammatory pathways rather than exerting broad, uniform suppression. In addition, the mRNA levels of chemokines such as *CCL5* and *CXCL9* were significantly modulated in the presence of OTC, which are known to recruit immune cells and contribute to pathogen clearance. Moreover, the RNA-seq results revealed that the TLRs signaling pathway, particularly involving TLR2 and TLR4, was significantly enriched. These TLRs are known to recognize Gram-positive bacteria and activate downstream NF-κB and MAPK pathways. Coincidentally, one of the prior studies showed that OTC protein could reduce the *L. monocytogenes*-induced inflammation in RAW 264.7 cells via TLR-2 mediated NF-kB and MAPK pathways [11]. Similarly, Cai et al. found that SlpA from *Lactobacillus acidophilus* CICC 6074 markedly attenuated inflammatory responses by reducing the production of mediators such as iNOS and COX-2, together with pro-inflammatory cytokines. This effect was suggested to involve the suppression of TLR4-dependent MAPK and NF-κB pathways, as well as NOD2- and NLRP3-associated intracellular signaling [21].

Activation of inflammation-associated pathways, such as NF-κB, can further stimulate barrier-regulatory pathways including MLCK, ultimately causing reduced expression of mucus-related proteins and altered distribution of tight-junction components [22,23]. The findings showed that wild-type *E. faecium* WEFA23 enhanced the expression of key TJ proteins, including Occludin and ZO-1, which were significantly reduced in the OTC-deficient strain. This suggested that OTC may contribute to the reinforcement of epithelial tight junctions, thereby limiting the translocation of *L. monocytogenes*. Similar barrier-protective effects have been reported in other surface layer proteins from lactic acid bacteria, such as SlpA and SlpB, which modulate epithelial permeability and immune activation [24,25,26]. Further, RNA-seq analysis revealed significant DEGs associated with the calcium channel signal pathway, including *ORAI3*, which is known to mediate store-operated calcium entry (SOCE) in epithelial cells, was down-regulated in the presence of OTC protein. It is reported that calcium signaling plays a pivotal role in maintaining intestinal epithelial barrier integrity, primarily through the regulation of tight junction (TJ) proteins such as Occludin, Claudins, and ZO-1 [27,28]. Additionally, it is particularly relevant in the context of *L. monocytogenes* infection, as the pathogen has been reported to exploit host calcium signaling to facilitate its internalization and translocation across the epithelial barrier [29]. These findings suggest that OTC may indirectly strengthen epithelial barrier function by regulating calcium signaling pathways. Therefore, modulation of calcium channel activity by probiotic components like OTC protein could represent an important mechanism underlying their protective effect against enteric pathogens. Moreover, transcriptomic analysis further identified several DEGs, such as *ADCY2* involved in the GABAergic synapse. Notably, *ADCY2* encodes adenylate cyclase, a key enzyme in cAMP signaling, which may be involved in epithelial cell signal transduction during host-microbe interaction [30]. The involvement of the GABAergic signaling pathway observed in the study may hold physiological relevance for intestinal epithelial regulation. Emerging evidence indicates that GABAergic components, including GABA receptors and metabolic enzymes, participate in maintaining epithelial homeostasis, modulating cellular stress responses, and contributing to barrier function and host-microbe interactions [31]. These findings suggested that activation of the GABAergic pathway may reflect an epithelial regulatory mechanism engaged during microbial challenge. However, the interpretation of this pathway and other transcriptomic outcomes should be considered in light of the limitations of the Caco-2 monolayer model used in this study. As a transformed, single-cell-type epithelial system, Caco-2 cells lack the cellular diversity, mucus layer, immune communication, and microenvironmental complexity of the native intestine [32]. Their restricted immune competence and absence of cross-talk with stromal or neuronal elements may influence how signaling pathways, including GABAergic signaling, are represented compared with in vivo conditions. These inherent limitations underscore the need for future validation using more physiologically relevant systems, such as organoids or in vivo models.

*L. monocytogenes* expresses a range of virulence genes that are essential for its adhesion, invasion, intracellular survival, and systemic dissemination [33,34]. In addition to modulating host responses and TJPs, OTC also exerted indirect effects on the pathogen itself. The results suggested that the mRNA levels of *plcA*, *plcB*, and *prfA* were significantly down-regulated by the wild-type strain rather than the mutant strain, possibly by interfering with pathogen-host cell interactions or altering the local micro-environment. This highlights the potential of probiotic-derived components not only in enhancing host defense but also in diminishing pathogen aggressiveness at the epithelial interface. Liu et al. highlighted the function of probiotic metabolites by using the cell-free supernatant of lactic acid bacteria-fermented milk, and the results showed that they could simultaneously downregulate the expression of key virulence genes of *Salmonella Dublin*, ETEC *E. coli* F5, and *Clostridium perfringens*, demonstrating the potential as a preventive for neonatal diarrhea in calves, potentially reducing antibiotic use [35]. Likewise, Fan et al. showed that CoQ0 markedly suppressed the transcription of *L. monocytogenes* genes involved in adhesion and invasion (*actA*, *inlA*, *inlB*, *plcA*, *plcB*, and *prfA*), reducing their expression by at least 60% relative to the control [36].

## 5. Conclusions

Despite the promising results, this study has several limitations. First, the findings were based on an in vitro Caco-2 cell model, which may not fully capture the complexity of the in vivo intestinal environment. Future studies using animal models are necessary to validate the protective effects of OTC against *L. monocytogenes* infection in a physiological context. Second, the exact molecular interaction between the OTC protein and host epithelial receptors remains to be elucidated. Identifying the specific host binding targets of OTC would greatly advance the understanding of its mode of action. Finally, future work should also consider purified OTC assays, phenotypic validation of OTC expression, inclusion of cell viability assessments, and more detailed statistical analyses to further strengthen mechanistic and functional evaluations.

**Figure 7:**
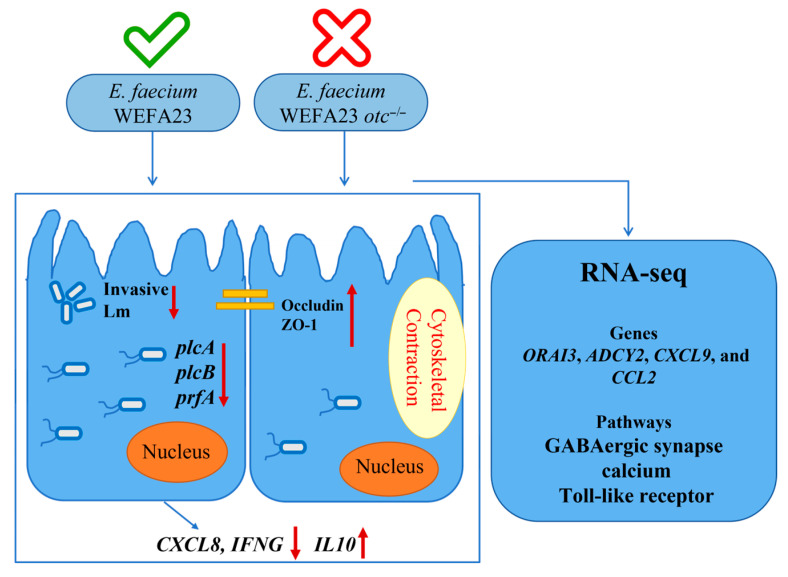
Diagram showing the pivotal role of OTC protein from *E. faecium* WEFA23 in anti-*L. monocytogenes* invasion in epithelial cells.

Collectively, the surface layer protein OTC from *E. faecium* WEFA23 plays a pivotal role in defending intestinal epithelial cells against *L. monocytogenes* invasion by enhancing tight junction integrity and modulating expression of inflammatory cytokines and virulence factors of the pathogen. Mechanistically, the activity involved *ORAI3*, *ADCY2*, *CCL5*, and *CXCL9* genes associated with calcium-related, GABAergic synapse and toll-like receptor signal pathways (Figure 7). These findings not only elucidate the functional mechanism of a novel probiotic component but also provide a theoretical foundation for the development of new probiotic intervention strategies.

## Figures and Tables

**Table 1:** Primers used in the study.

Name	Forward Primer (5′-3′)	Reverse Primer (5′-3′)
*IL6*	AGCAAAGCAAAGAAACCGAT	CAGCTCTGAGATGGCTTCAG
*IL1B*	TACAGTGGCAATGAGGAT	ATGAAGGGAAAGAAGGTG
*TNF*	TTTGATCCCTGACATCTGGA	GGCCTAAGGTCCACTTGTGT
*IFNG*	AGGCTTTATCTCAGGGGCCA	AGCACTGGCTCAGATTGCAG
*IL10*	AAGCCTGACCACGCTTTCTA	TCCGAGACACTGGAAGGTGA
*CXCL8*	ACCCCAAGGAAAACTGGGTG	GTTTGCTGTGCTTCTCTTGGA
*TJP1*	AGCCTTGCAAAGCCAGCTCA	AGTGGCCTGGATGGGTTCATAG
*OCLN*	AAGAGTTGACAGTCCCATGGCATAC	ATCCACAGGCCAAGTTAATGGAAG
*CLDN1*	GCATGAAGTATATGAAGTGCTTGGA	CGATTCTATTGCCATACCATGCTG
*ACTB*	GGCTATCCAGCGTACTCCAAA	CGGCAGGCATACTCATCTTTTT
*prfA*	TGAGCAAGAATCTTACGCACTTTT	GCTAGGCTGTATGAAACTTGTTTTTG
*actA*	CGGGTAAATGGGTACGTGAT	TGGTCAATTAACCCTGCACTT
*plcA*	TCGGACCATTGTAGTCATCTTGA	CACAAATTCGGCATGCAGTT
*plcB*	CGCAGCTCCGCATGATATT	GATTATCCGCGGACCAACTAAG
*inlA*	AATGTAACAGACACGGTCTCACAAA	TCCCTAATCTATCCGCCTGAAG
16 S rRNA	AGAGTTTGATCCTGGCTCAG	GGCTACCTTGTTACGACTT
*ORAI3*	CATTTTGGGGGAAGATTTCG	GTAGAAACACCCAAATCCCT
*ADCY2*	GCCGTGTTCAACATGTTTTT	CCACCTGATTATTTGAGGCT
*CCL5*	ATGCTTGGTTGCTATTTTGG	CAGTAGCAATGAGGATGACA
*CXCL9*	TGATTGGAGTGCAAGGAAC	GCTGAATCTGGGTTTAGACA

## Data Availability

The original contributions presented in the study are included in the article, further inquiries can be directed to the corresponding author.
